# Gonadotropins in Mini-Puberty: Pathophysiological and Therapeutic Implications for Male Congenital Hypogonadism

**DOI:** 10.3390/children13010133

**Published:** 2026-01-15

**Authors:** Ignazio Cammisa, Donato Rigante, Clelia Cipolla

**Affiliations:** 1 Department of Life Sciences and Public Health, Fondazione Policlinico Universitario A. Gemelli IRCCS, 00168 Rome, Italy; donato.rigante@unicatt.it (D.R.); clelia.cipolla@policlinicogemelli.it (C.C.); 2 Università Cattolica Sacro Cuore, 00168 Rome, Italy

**Keywords:** mini-puberty, congenital hypogonadism, hypothalamic–gonadal axis, gonadotropins, hormonal therapy, children, personalized medicine

## Abstract

**Background:** Mini-puberty is a transient but critical postnatal activation of the hypothalamic–pituitary–gonadal axis, essential for male gonadal maturation, penile and testicular growth, and future reproductive potential: this physiological hormonal surge is absent or blunted in congenital hypogonadotropic hypogonadism (CHH), often manifesting as micropenis, cryptorchidism, and impaired Sertoli cell proliferation. **Objective:** The aim of this review is to summarize current evidence on the impact of early gonadotropin therapy in male infants with CHH. **Methods:** We conducted a comprehensive literature review using PubMed, including studies reporting on male infants with confirmed or suspected CHH receiving gonadotropin therapy. Keywords included “mini-puberty and hypogonadism”, “gonadotropins and infancy,” and “gonadotropin therapy in CHH.” Eligible studies reported biochemical outcomes (luteinizing hormone, follicle-stimulating hormone, testosterone, inhibin B, anti-Müllerian hormone) and clinical measures (penile length, testicular volume, testicular descent). Data extraction focused on endocrine responses, genital growth, and safety. **Results:** Twelve studies including 95 infants were analyzed. Early gonadotropin therapy effectively restored postnatal hormonal levels, with consistent increases in testosterone, inhibin B, and anti-Müllerian hormone. Clinically, treatment induced significant penile growth, increased testicular volume and partial or complete testicular descent in the majority of cases. Both continuous infusion and intermittent injection regimens were effective, though hormone kinetics and growth responses varied. No serious adverse events were reported, and therapy was generally well tolerated. **Conclusions**: Early gonadotropin therapy during mini-puberty represents a safe and effective intervention to replicate the physiological postnatal hormonal surge in male infants with CHH. Prospective longitudinal studies are warranted to evaluate sustained effects on puberty, fertility, and adult reproductive function.

## 1. Introduction

Mini-puberty is a brief but physiologically critical period of hypothalamic–pituitary–gonadal (HPG) axis activation in early postnatal life [1]. In healthy male infants, luteinizing hormone (LH) and follicle-stimulating hormone (FSH) rise shortly after birth, peaking between 4 and 10 weeks, together with a transient increase in testosterone that declines to prepubertal levels by around 6 months [1,2,3]. Despite its short duration, mini-puberty plays a key organizational role in male reproductive development: FSH drives Sertoli cell proliferation, thereby establishing future spermatogenic potential, while LH-induced testosterone production supports genital development, testicular descent, and increases in testicular volume [4,5,6,7,8]. These mechanisms provide a strong biological rationale for early gonadotropin therapy in infants with congenital hypogonadotropic hypogonadism (CHH), due to impaired gonadotropin-releasing hormone (GnRH) secretion or action, in whom the normal postnatal hormonal surge is absent or blunted [1,2,3,8,9]. This deficiency may lead to early genital abnormalities and long-term consequences for testicular development and spermatogenic potential [10,11,12]. Restoring mini-puberty through timely gonadotropin treatment may help optimize testicular maturation, improve future responsiveness to fertility therapies, and mitigate lifelong reproductive and systemic consequences. Accordingly, mini-puberty is increasingly recognized as a critical diagnostic and therapeutic window [13,14,15,16].

The primary aim of this review is to summarize the current evidence on gonadotropin therapy in male infants with CHH during mini-puberty, highlighting its physiological rationale, clinical outcomes, and potential long-term benefits.

## 2. Materials and Methods

We conducted a comprehensive literature review to investigate the role of gonadotropin therapy during mini-puberty in male infants with CHH.

### 2.1. Inclusion and Exclusion Criteria

To be included in this review, studies were required to meet the following criteria: (1) involvement of male infants undergoing either physiological and endocrinological evaluation of mini-puberty with a confirmed diagnosis of hypogonadism; and (2) exposure to hormonal therapy with gonadotropins. Studies were excluded if they were not published in English, focused exclusively on adult populations, or lacked endocrine assessments relevant to mini-puberty or gonadotropin therapy.

### 2.2. Search Strategy

**Figure 1:**
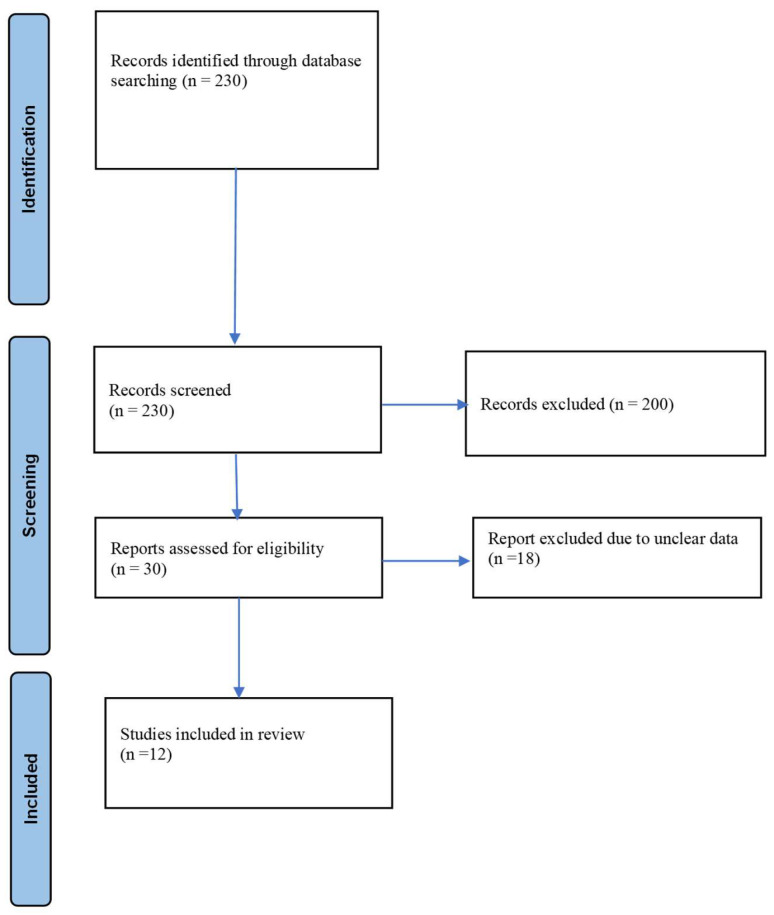
PRISMA 2020 flow diagram for new systematic reviews which included searches of databases.

We conducted a scoping review without date restrictions using the PubMed bibliographic database. The search strategy included the following terms: “mini-puberty and hypogonadism”, “mini-puberty and gonadotropins”, “gonadotropins and congenital hypogonadism”, “gonadotropins and children”, “gonadotropins and infancy”, and “mini-puberty and hormonal therapy”. The review was performed in accordance with the Preferred Reporting Items for Systematic Reviews and Meta-Analyses (PRISMA) guidelines to ensure transparency and methodological rigor. Abstracts were independently screened by two reviewers (IC and CC) based on predefined inclusion and exclusion criteria to assess eligibility for full-text evaluation. All studies deemed eligible were subsequently reviewed in full. Any discrepancies between reviewers were resolved through open discussion and consensus. Both randomized and non-randomized controlled trials were included, along with observational studies of any design, including prospective and retrospective cohort studies, case–control studies, and cross-sectional analyses. In addition, case series and individual case reports were considered. A detailed description of the search strategy is provided in Figure 1.

### 2.3. Study Selection

A total of 230 records were retrieved from the PubMed database. During the initial screening, 30 articles published in languages other than English, 60 records lacking accessible full texts, and 90 duplicate entries were excluded. In the subsequent screening phase, titles and abstracts were evaluated, leading to exclusion of 20 additional records that did not satisfy the predefined inclusion criteria. Of the 30 studies remaining, 18 were excluded following further assessment due to concerns regarding data reliability. Ultimately, 12 studies met the eligibility criteria and were included in the review. The study selection process is summarized in the PRISMA flow diagram, and the main findings are reported in Table 1.

### 2.4. Data Extraction

Data extraction was conducted under the supervision of the senior investigator (CC). For each study meeting the inclusion criteria we systematically collected information on study design, demographic and clinical characteristics of the study population, gonadotropin therapy protocols, and biochemical and clinical outcomes. When available, study limitations and declared conflicts of interest were also recorded. The study was prepared in accordance with the Preferred Reporting Items for Systematic Reviews and Meta-Analyses extension for Scoping Reviews (PRISMA-ScR) statement. The review protocol was not registered in any database. As this review was based exclusively on previously published data, formal ethical approval was not required. Results were synthesized using a narrative approach to describe and contextualize the evidence emerging from the included studies.

## 3. Results

This review comprises 12 studies, the majority of which are case series or case reports, including a total of 95 patients [17,18,19,20,21,22,23,24,25,26,27,28]. Most patients were affected by CHH. Specifically, 65/95 (68.42%) had CHH, 18/95 (18.95%) had multiple pituitary hormone deficiency (MPHD), 6/95 (6.32%) had Kallmann syndrome, 2/95 (2.11%) had CHARGE syndrome, 4/95 (4.21%) had septo-optic dysplasia, 1/95 (1.05%) had Prader–Willi syndrome, 1/95 (1.05%) had trisomy 21, and 1/95 (1.05%) had partial androgen insensitivity syndrome. Across the included studies, early postnatal administration of gonadotropins consistently induced biochemical and clinical features of mini-puberty in male infants. At baseline, gonadotropin and testosterone levels were uniformly low or undetectable. Following treatment, serum testosterone increased markedly across all series, reaching physiological or supraphysiological concentrations. Testosterone increased from undetectable levels to mean concentrations of 3.3–3.5 ng/mL in short-term protocols, and up to supraphysiological levels in some cohorts. In larger comparative cohorts testosterone levels rose significantly with both continuous pump and injection-based regimens, with a higher daily increment observed in injection protocols (+0.04 vs. +0.01 ng/mL/day). Inhibin B levels increased significantly across studies, with mean or median rises ranging from approximately 100 to 300 pg/mL. Anti-Müllerian hormone (AMH) concentrations similarly increased in most cohorts, particularly in studies employing combined LH/FSH administration or continuous infusion regimens. However, in one long-term follow-up study, inhibin B levels declined, suggesting a potentially transient effect of early FSH exposure. Penile growth represented one of the most consistent clinical responses to treatment. Stretched penile length (SPL) increased significantly in all studies, with gains ranging from a median of 12–25 mm in short-term protocols to increases from approximately 1.0–2.0 cm at baseline to 3.0–4.5 cm post-treatment. In comparative analyses, penile growth occurred more rapidly with injection-based regimens than with pump-based regimens (+0.16 vs. +0.10 mm/day). Testicular volume (TV) increased from sub-prepubertal values (<1 mL) to approximately 1–2 mL in most series, with ultrasound-based assessments demonstrating relative increases of 150–170% in some reports. Although testicular length and width did not always change significantly, volumetric gains were documented, particularly in studies using continuous gonadotropin infusion. Testicular descent improved substantially with therapy: complete scrotal descent was achieved in 80–100% of treated infants in several case series, often within the first 1–3 months of treatment. In larger cohorts, the risk of persistent cryptorchidism decreased significantly over time (OR ≈ 0.97 per day), with comparable effects across treatment modalities. In some cases gonadotropin therapy reduced or obviated the need for surgical orchiopexy. No serious adverse events were reported across the included studies with gonadotropin therapy, which was generally well tolerated, even when administered continuously for up to six months. However, there are no available data regarding long-term safety with limited evidence to short-term endocrine outcomes.

## 4. Discussion

**Figure 2:**
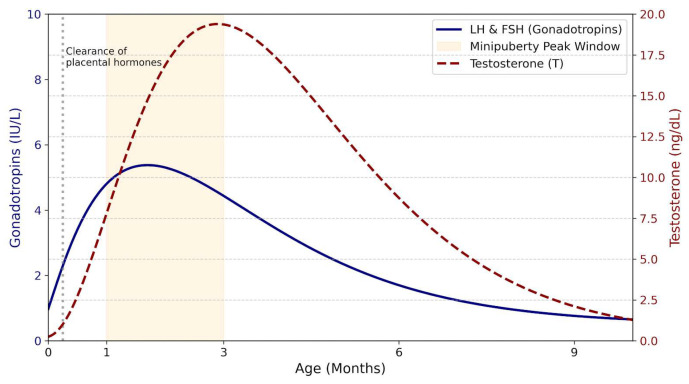
Hormonal profiles during mini-puberty in male infants.

Mini-puberty represents a brief but critical developmental window during early postnatal life, characterized by transient activation of the HPG axis and increased gonadal activity. It constitutes the second major phase of HPG axis activation, following fetal activation in utero and preceding reactivation at puberty [12,29]. During the early postnatal period, pulsatile hypothalamic secretion of GnRH progressively increases, resulting in enhanced secretion of LH and FSH by pituitary gonadotroph cells. This hormonal surge typically peaks at approximately one month of age and plays a pivotal role in early gonadal maturation and sex steroid production [12,29]. At birth, LH and FSH secretion is suppressed by placental hormones, which are cleared within the first week of life [12]. The subsequent release of this inhibition leads to a postnatal rise in gonadotropin levels lasting up to 6–9 months, with a marked increase in the LH/FSH ratio during mini-puberty, clearly distinguishing male from female infants. In boys, this surge is associated with increased testosterone production by Leydig cells and enhanced secretion of inhibin B and AMH by Sertoli cells (Figure 2). Testosterone concentrations peak between 1 and 3 months of age and then decline to low or undetectable levels by 4–6 months [30,31].

Although proliferation of both Leydig and Sertoli cells occurs during infancy, the lack of androgen receptor expression in Sertoli cells prevents the initiation of spermatogenesis and suppression of AMH production, resulting in persistently high AMH levels despite elevated testosterone concentrations [3,30,31]. Accordingly, mini-puberty is considered a crucial phase for male gonadal development, acting as a developmental “priming” period that programs the testes for later normal function in life. It is associated with key physiological processes of gonadal maturation, including penile and testicular growth and proliferation of gonadal cells [32]. Penile length has been shown to correlate positively with serum testosterone levels, increasing from birth through early childhood, with the highest growth velocity observed within the first three months of life (approximately 1 mm per month) [32,33]. In contrast, TV increases significantly during the first 5–6 months of life, rising from approximately 0.27 to 0.44 cm^3^, before declining to around 0.31 cm^3^ by nine months of age. This developmental pattern correlates positively with circulating FSH concentrations and is likely driven by Sertoli cell proliferation within the seminiferous tubules [32,33,34]. The absence of mini-puberty may therefore have both short- and long-term consequences and should be regarded as an early warning sign of CHH. CHH is a rare and predominantly male disorder caused by impaired GnRH secretion or action, resulting in deficient pituitary LH and FSH production. It may occur in isolation or in association with combined pituitary hormone deficiencies and is most often of genetic origin [29]. In male infants, CHH should be suspected in the presence of micropenis and/or cryptorchidism identified at birth. These early clinical features warrant prompt endocrine evaluation to confirm the absence of a physiological gonadotropin surge during mini-puberty, with additional congenital anomalies potentially supporting the diagnosis [29,35]. Male neonates with CHH exhibit persistently low circulating levels of LH, FSH, testosterone, AMH, and inhibin B throughout the mini-puberty window, which extends from approximately the second week of life to six months of age. The absence of this expected postnatal hormonal rise substantiates the clinical suspicion of CHH raised by abnormalities of the external genitalia [36,37]. The optimal time for measuring reproductive hormone concentrations is between 4 and 8 weeks of life, although a reliable assessment can be performed up to six months of age [38]. Over the past two decades, multiple studies have highlighted the association between disrupted mini-puberty and hypogonadotropic hypogonadism, particularly in males, consistently demonstrating that the condition is characterized by the absence of the physiological postnatal surge in FSH, LH, and testosterone. Mini-puberty thus represents a unique and time-limited window of opportunity for early diagnosis and treatment of CHH. Although testosterone replacement therapy is widely used in males with CHH to induce virilization and secondary sexual characteristics, it does not promote testicular development or preserve fertility potential. In contrast, gonadotropin therapy administered during mini-puberty may support testicular maturation, enhance potentially future fertility potential, and facilitate testicular descent, potentially reducing the need for surgical intervention [29]. Infertility in CHH results from impaired spermatogenesis due to LH and FSH deficiency, and responsiveness to gonadotropin therapy in adulthood is highly variable, being poorer in individuals with history of cryptorchidism, reduced testicular volume, and low inhibin B concentrations [29,39]. Baseline inhibin B has emerged as a key predictor of spermatogenic outcomes, with higher levels associated with better treatment response. In this context, the absence of mini-puberty has been linked to reduced Sertoli cell responsiveness and inferior fertility outcomes, highlighting the potential long-term importance of early postnatal HPG axis activation. Accordingly, combined gonadotropin therapy designed to recapitulate mini-puberty in male infants with CHH may promote Sertoli cell proliferation and improve testicular development during a critical developmental window [29,39,40].

One of the most consistent findings across all included studies is the robust activation of the HPG axis following exogenous gonadotropin administration. In untreated infants with CHH or related central deficiencies, baseline levels of LH, FSH, and testosterone were uniformly low or undetectable, reflecting the absence of endogenous GnRH-driven stimulation. Gonadotropin therapy—administered via either continuous infusion or intermittent injections—effectively restored this hormonal environment, resulting in testosterone concentrations within or above the physiological range observed during normal mini-puberty. For example, Bougnères et al. [18] reported increases from undetectable levels to 7.6 and 5.2 nmol/L after six months of continuous infusion, Stoupa et al. [21] observed rises from undetectable to 3.5 ± 4.06 ng/mL, Mesas-Aróstegui et al. [26] documented supraphysiological testosterone concentrations following combined HCG and recombinant FSH therapy, and Roddick et al. [28] reported an increase from 0 ± 0.1 nmol/L to 22 ± 5.6 nmol/L. The magnitude of testosterone increase varied according to treatment modality, dosage, and duration. Injection-based regimens generally induced a more rapid daily rise compared with pump-based protocols, whereas continuous infusion better replicated physiological hormone exposure. Although the clinical significance of transient supraphysiological testosterone peaks in infancy remains unclear, no study reported serious adverse effects related to androgen excess, indicating a favorable short-term safety profile. Beyond androgen replacement, a key advantage of gonadotropin therapy over testosterone monotherapy is its ability to stimulate Sertoli cell function via FSH exposure. Across the included studies, markers of Sertoli cell activity, namely inhibin B and AMH, consistently increased following treatment, often reaching age-appropriate or high-normal levels. For instance, Main et al. [17] reported rises in inhibin B from 121 to 268 pg/mL and in AMH from 40 to 55 pmol/L; Bougnères et al. [18] observed normalization of both inhibin B and AMH after six months of continuous infusion. Stoupa et al. [21] documented increases in inhibin B from 94.8 ± 74.9 to 469.4 ± 282.5 pg/mL (*p* = 0.04) and in AMH from 49.6 ± 30.6 to 142 ± 76.5 ng/mL (*p* = 0.03), while Papadimitriou et al. [22] reported rises from subnormal to normal levels (inhibin B from 27.8 to 365 pg/mL; AMH from 1.54 to 150 ng/mL). Similar robust responses were observed in later series, including Mesas-Aróstegui et al. [26] and Roddick et al. [28]. These findings are particularly relevant, as Sertoli cell proliferation during early life is a major determinant of adult spermatogenic potential [29]. Experimental and clinical data indicate that the final Sertoli cell number is largely fixed by the end of puberty, and early deficits may irreversibly limit sperm output. By promoting Sertoli cell proliferation during mini-puberty, gonadotropin therapy may therefore exert long-lasting organizational effects on the testis [29,39]. Although direct evidence linking early treatment to improved adult fertility is limited, the consistent biochemical responses observed across studies strongly support this potential benefit. Notably, one long-term follow-up study reported a decline in inhibin B levels despite early treatment, raising the possibility that the beneficial effects of neonatal FSH exposure may be transient [23]. This observation underscores the need for long-term longitudinal studies to determine whether early gonadotropin therapy confers a sustained advantage or primarily serves as a priming intervention that enhances responsiveness to pubertal induction and in adolescence or adulthood.

From a clinical perspective, penile growth emerged as one of the most consistent and reproducible outcomes across all studies. Significant increases in SPL were observed regardless of treatment modality. For example, Main et al. [17] reported an increase from 1.6 to 2.4 cm, Bougnères et al. [18] observed gains from 8 to 30 mm and from 12 to 48 mm, and Stoupa et al. [21] documented increases from 13.8 ± 4.5 to 42.6 ± 5 mm (*p* < 0.0001). Similar improvements were noted in Papadimitriou et al. [22] (from 2 to 3.8 cm), Mesas-Aróstegui et al. [26] (from 20 to 44 mm; *p* = 0.008), and Roddick et al. [28] (from 1.0 ± 0.3 to 3.0 ± 0.4 cm; *p* < 0.001). These increases are often sufficient to normalize penile size relative to age-matched peers, addressing both functional and psychosocial implications associated with micropenis, which may persist despite later androgen therapy if not treated early. Testicular growth, while generally more modest than penile growth, was consistently documented. TV increased from sub-prepubertal values (<1 mL) to approximately 1–2 mL in most series, reflecting both Leydig and Sertoli cell activation. Ultrasound-based measurements proved more sensitive in detecting these volumetric changes. Reported increases included Main et al. [17] (TV + 170%), Bougnères et al. [18] (from 0.45 to 2.10 mL), Sarfati et al. [19] (from 0.33 to 2.3 mL), Lambert et al. [20] (from 0.43 to 1.64 mL, *p* < 0.001), and Roddick et al. [28] (from 0.156 ± 0.09 to 0.296 ± 0.31 mL). Several studies reported complete scrotal descent in the majority of treated infants, often within the first 1–3 months of therapy. For instance, Papadimitriou et al. [22] documented descent in all treated testes, and Roddick et al. [28] reported descent in 4/5 infants. In larger cohorts, early gonadotropin therapy significantly reduced the risk of persistent cryptorchidism and, in some cases, eliminated the need for surgical orchiopexy. Given the established association between cryptorchidism, impaired spermatogenesis, and increased malignancy risk, these results suggest that early postnatal hormonal intervention may confer both short- and long-term clinical benefits beyond cosmetic outcomes.

One of the most debated aspects of postnatal gonadotropin therapy is the optimal mode of administration. The studies reviewed employed diverse therapeutic strategies, including continuous subcutaneous infusion of recombinant LH (r-LH)/FSH (r-FSH), intermittent subcutaneous injections of LH/FSH or human chorionic gonadotropin (HCG) combined with FSH, and, in selected cases, GnRH pump therapy. Hormone replacement therapy in this context is tailored to the differing half-lives of gonadotropins. LH has a short half-life, necessitating either multiple daily administrations or pulsatile delivery via continuous subcutaneous infusion pump, whereas HCG’s longer half-life allows for less frequent subcutaneous injections. rhLH or HCG can be administered concurrently with recombinant FSH rhFSH. The use of HCG is based on its action through the LH/choriogonadotropin receptor, which stimulates Leydig cell activity and enhances intratesticular testosterone production [41]. Prior to approximately 4–5 years of age, Sertoli cells do not express androgen receptors; therefore, unlike in adolescents, combining rhFSH with rhLH or HCG in infants promotes Sertoli cell proliferation without inducing differentiation [8]. All approaches were effective in inducing mini-puberty-like biochemical and clinical changes, but differences in hormonal kinetics and clinical outcomes were evident. Continuous infusion regimens appeared to more closely replicate physiological gonadotropin exposure and were associated with robust Sertoli cell activation. For example, Bougnères et al. [18] used continuous LH/FSH infusion for six months in infants aged 2–5 months, achieving normalization of inhibin B and AMH, alongside increased TV. Similarly, Lambert et al. [20] reported substantial rises in LH, FSH, testosterone, inhibin B, and AMH with continuous infusion over approximately six months, with mean SPL increasing from 20.2 to 37.4 mm and TV from 0.43 to 1.64 mL (*p* < 0.001). Injection-based regimens, in contrast, often produced more rapid rises in testosterone and faster penile growth, though Sertoli cell markers also improved. Papadimitriou et al. [22] administered daily LH/FSH injections for three months in infants averaging 4.2 months of age, leading to normalization of LH, FSH, inhibin B, AMH, and testosterone, with SPL increasing from a median of 2 to 3.8 cm and all testes achieving scrotal descent. Mesas-Aróstegui et al. [26] reported that discontinuous HCG plus recombinant FSH-alpha injections over a median of 3.5 months produced supraphysiological testosterone levels, significant SPL increases from 20 to 44 mm, and notable TV gains (*p* = 0.005). These data suggest that while both continuous and intermittent administration can effectively reproduce mini-puberty-like hormonal and anatomical responses, the kinetics of androgen rise and penile growth differ between strategies. Therefore, treatment should be individualized according to clinical priorities, feasibility, and family preferences. Importantly, initiation of therapy within the first months of life—typically between 1 and 6 months—reinforces the concept that mini-puberty represents a narrow, yet highly responsive, therapeutic window.

**Figure 3:**
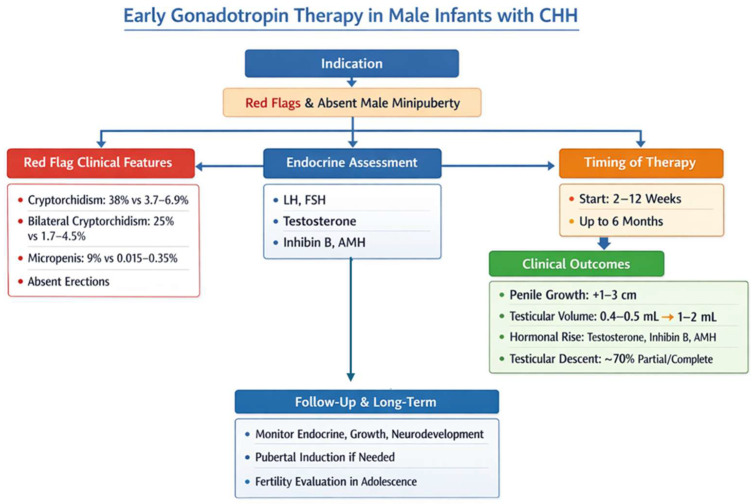
Proposed diagnostic and therapeutic algorithm in the case of suspected congenital hypogonadotropic hypogonadism (CHH).

Early gonadotropin therapy in male infants with CHH is indicated for those with red flags to suggest CHH [42,43]. The proposed indicators are generally apparent at or shortly after birth and include cryptorchidism (observed in 38% of affected infants vs. 3.7–6.9% in the general population at birth), bilateral cryptorchidism (25% vs. 1.7–4.5%), micropenis (9% vs. 0.015–0.35%), and absent erections during diaper changes [37]. Additional non-reproductive phenotypes associated with CHH include cleft lip and/or palate (5% vs. 0.1%) and hearing impairment (6% vs. 0.12%) [37]. Infants presenting these “red flag” features should undergo prompt endocrine assessment to evaluate LH, FSH, testosterone, inhibin B, and AMH levels, confirming the diagnosis and guiding treatment. Therapy is ideally started between 2 and 12 weeks of age, although benefits have been reported up to six months. Clinical outcomes consistently include significant penile growth (1–3 cm over 3–4 months), increased testicular volume (0.4–0.5 mL to 1–2 mL), robust rises in testosterone, inhibin B, and AMH, and partial or complete testicular descent in approximately 70% of cases. Follow-up should monitor endocrine function, testicular growth, and neurodevelopment. We included a proposed management algorithm based on our experience at our center, as illustrated in Figure 3, to provide a practical framework for gonadotropin therapy in infants with CHH.

## 5. Limitations

Despite encouraging preliminary results, evidence on early gonadotropin therapy in male infants with CHH is limited by several factors. Studies are highly heterogeneous, differing in type of gonadotropins used (FSH + LH, FSH + HCG, or pulsatile GnRH), dosing regimens (pump vs. injection), timing of initiation (from the first weeks to several months of life), and duration of therapy. Most reports are small case series and cohorts, often fewer than 10–20 infants, restricting statistical power and generalizability with low level of evidence. The inclusion of case series and case reports from specialized centers carries a high risk of publication and referral bias, lacks untreated control groups, and may lead to an overestimation of treatment effects. The inclusion of patients with conditions pathophysiologically distinct from CHH, such as partial androgen insensitivity syndrome and trisomy 21, represents an additional limitation, as the lack of detailed patient-level data in the original studies precluded their exclusion or separate analysis and may have reduced the conceptual specificity of our findings. No study reports adult reproductive outcomes, such as spermatogenesis, paternity, or the need for pubertal induction; the limited long-term follow-up available suggests a possible decline in inhibin B, raising the concern that the effects of neonatal FSH exposure may be transient. Interpretation of results is further constrained by unaddressed confounders, including the lack of baseline testicular histology, incomplete genetic characterization, and the potential influence of concomitant cryptorchidism surgery. Moreover, ethical, practical, and logistical challenges, including early identification of CHH, timely hormone measurements, neonatal pump management, and lack of standardized regimens, complicate implementation. Critically, although the theoretical rationale for early intervention is strong, definitive evidence that mini-puberty induction improves adult fertility remains insufficient. Given the absence of long-term and definitive outcome data, the use of gonadotropin therapy in infants with CHH should always be carefully discussed with parents or caregivers, with informed consent obtained, and with clear emphasis on the short-term therapeutic objectives in the context of limited long-term evidence.

Overall, while early gonadotropin therapy shows promise, protocol heterogeneity and limited long-term data hinder a proper development of standardized treatment guidelines.

## 6. Conclusions

Mini-puberty constitutes a crucial developmental period in male infants, necessary for appropriate gonadal maturation and establishment of future reproductive potential. In CHH the lack of this postnatal activation of the HPG axis results in persistently low levels of testosterone, LH, FSH, inhibin B, and AMH, clinically presenting as micropenis and/or cryptorchidism. Early recognition of these “red flag” signs, followed by prompt endocrine evaluation, is therefore essential for swift recognition and intervention. Administration of exogenous gonadotropins during mini-puberty effectively recreates the physiological postnatal hormonal surge, stimulating testosterone synthesis, Sertoli cell proliferation, penile and testicular growth, and facilitating testicular descent. Both continuous infusion and intermittent injection protocols have proven effective, with variations in hormone kinetics and clinical outcomes supporting individualized treatment approaches. Importantly, these therapies are generally well tolerated, with no serious adverse events reported in the short-term. In summary, early gonadotropin therapy not only addresses immediate genital anomalies but also holds the potential to improve long-term reproductive outcomes. Prospective longitudinal studies are needed to assess whether neonatal intervention confers sustained benefits into adolescence and adulthood, particularly regarding spermatogenic capacity and response to pubertal induction. Incorporation of early hormonal biomarkers into clinical practice may further enhance patient selection, optimize treatment timing, and ultimately improve the natural history of CHH.

## Figures and Tables

**Table 1:** List of studies related to gonadotropin therapy in male infants with hypogonadism.

Study	Study Design	Sample Size (N) and Population	Treatment	Mean Age (for Starting Therapy)	Biochemical Outcomes	Clinical Outcomes
Main et al. (2002) [17]	Case reports	1 CHH	Recombinant human LH 20 IU and FSH 21.3 IU SC twice weekly	7.9 months	LH, FSH, inhibin B and estradiol increased to values within normal limits (0.7–1.88 IU/L, 0.17–3.24 IU/L, 121–268 pg/mL and 40–55 pmol/L, respectively), whereas serum testosterone remained undetectable	SPL increased from 1.6 to 2.4 cm and testicular volume increased by 170%
Bougnères et al. (2008) [18]	Case reports	1 CHH 1 MPHD	Recombinant LH and FSH SC via infusion pump for six months	2–5 months	Mean testosterone increased from undetectable levels to 7.6 and 5.2 nmol/liter. Inhibin B and anti-Müllerian hormone increased to normal levels.	Mean TV increased from 0.45 to 0.57 mL at birth to 2.10 mL at 7 months. SPL increased from 8 to 30 mm and from 12 to 48 mm
Sarfati et al. (2015) [19]	Case reports	1 KS	Recombinant LH and FSH SC via infusion pump for six months	1 month	NA	Increased TV from 0.33 to 2.3 mL; increased SPL from 1.5 to 3.8 cm
Lambert et al. (2016) [20]	Case reports	5 CHH 3 CPHD	Recombinant LH and FSH SC via infusion pump for six months	6.03 months	Increased LH, FSH, testosterone, inhibin B for all patients; increased AMH for 7 of 10 patients.	Mean SPL increased from 20.2 mm to 37.4 mm (*p* < 0.001) in infants with CHH and from 22.6 mm to 44.3 mm (*p* < 0.001) in infants with CPHD; TV increased from 0.43 mL to 1.64 mL (*p* < 0.001)
Stoupa et al. (2017) [21]	Single center study	4 CHH 1 MPHD 1 PAIS	Recombinant LH and FSH SC via infusion pump for three-six months	4.2 months	In CHH, marked increases in serum testosterone concentrations (from undetectable levels to 3.5 ± 4.06 ng/mL [12.15 ± 14.09 nmol/L]), inhibin B (from 94.8 ± 74.9 to 469.4 ± 282.5 pg/mL; *p* = 0.04), and anti-Müllerian hormone (AMH) (from 49.6 ± 30.6 to 142 ± 76.5 ng/mL; *p* = 0.03).	SPL increased from 13.8 ± 4.5 to 42.6 ± 5 mm; *p* < 0.0001); in PAIS SPL increased from 13 to 38 mm
Papadimitriou et al. (2019) [22]	Case series	5 KS 2 CHH 1 CHARGE 2 SOD 1 MPHD	Daily SC injections of Pergoveris (LH/FSH 75/150 IU) for 3 months	4.2 months	Median LH and FSH, both undetectable before treatment, reached high normal levels of 4.45 IU/L and supranormal levels 83 IU/L, respectively; median inhibin-b and anti-Mullerian hormone levels increased from subnormal (27.8 and 1.54 ng/mL, respectively) to normal levels (365 and 150 ng/mL, respectively); median testosterone increased from just detectable (0.02 ng/mL) to normal levels (3.3 ng/mL)	SPL increased from a median of 2 to 3.8 cm. During therapy, all testes descended to the scrotal position.
Kohva et al. (2019) [23]	Retrospective cohort study	4 CPHD 1 CHARGE	r-hFSH (3.4 IU/kg–7.5 IU/kg per week in 2 or 3 SC doses for 3–4.5 months) combined with Testosterone (25 mg i.m. monthly for three months)	0.7–4.2 months	Inhibin B levels increased from 76 ± 18 ng/L to 176 ± 80 ng/L (*p* = 0.04); long-term follow-up data, available for three patients, showed no significant differences in inhibin B levels compared with untreated CHH controls	Mean SPL increased of 81 ± 50% (*p* = 0.04).
Avril et al. (2023) [24]	Multicenter retrospective study	35 CHH	Recombinant LH and FSH SC via infusion pump for three-six months or multiweekly SC injections of recombinant HCG and FSH for 3 months	5.1 months (pump group) 13 months (infusion group)	Mean testosterone level increased +0.04 ng/mL per day in the injection group vs. +0.01 ng/mL per day in the pump group, *p* = 0.001); serum AMH increased +3.6 pmol/L per day in the injection group vs. + 2.9 pmol/L per day in the pump group, *p* = 0.546); serum inhibin B levels increased +2.8 pg/mL per day in the injection group vs. +1.6 pg/mL per day in the pump group, *p* = 0.066)	SPL increased +0.16 ± 0.02 mm per day in the injection group vs. +0.1 ± 0.02 mm per day in the pump group, *p* = 0.002). No change in testicular length and testicular width during treatment and between the two groups.
Castro et al. (2024) [25]	Case series	5 CHH or CPHD	r-FSH combined with HCG or r-LH	0.4 years	Increased serum FSH levels (median rise: 22.7 IU/L; range: 0.0–55.0 IU/L), and inhibin B concentrations (median increase: 256 pg/mL; range: 36.9–605.1 pg/mL)	Increased SPL (median gain: 12.5 mm; range: 2.0–25 mm), TV (median increase: 0.3 cc; range: 0.1–1.13 cc)
Mesas-Aróstegui et al. (2024) [26]	Case series	4 CHH 4 MPHD 1 Prader–Willi	Discontinuous injections with SC HCG (62.5–500 IU) two times per week and recombinant FSH-alpha (37.5–75 IU) three times per week for a median of 3.5 months	1.50 months	Testosterone increased to supraphysiological levels	SPL increased from 20 mm (18.00–26.50) to 44 mm (40–46) in length (*p* = 0.008); all patients showed a significant increase in TV (*p* = 0.005; large effect size, r = 0.937)
Ren et al. (2025) [27]	Case series	8 CHH	GnRH pump or HCG combined with human gonadotropin for 1–3 months	6 months	Serum testosterone and inhibin-B levels increased from being undetectable to 737.1 ± 409.5 ng/dL and from 47.88 ± 23.03 to 168.94 ± 59.34 pg/mL, respectively	PL increased from 1.44 ± 0.69 to 3.48 ± 0.38 cm (*p* < 0.0001), and TV increased from <1 mL to 1–2 mL
Roddick et al. (2025) [28]	Case series	4 MPHD (2 with SOD) 1 Trisomy 21	HCG alfa (10–20 μg twice-weekly) and recombinant FSH (25–50 IU thrice-weekly) for 16 ± 6.3 weeks	11 ± 4.6 weeks	Testosterone increased from 0 ± 0.1 nmol/L to 22 ± 5.6 nmol/L (*p* < 0.001) and inhibin B rose from 76 ± 29 ng/L to 228 ± 148 ng/L (*p* = 0.02)	SPL increased from 1.0 ± 0.3 cm to 3.0 ± 0.4 cm (*p* < 0.001), TV increased from 0.156 ± 0.09 mL to 0.296 ± 0.31 mL (*p* = 0.09); testicular descent into scrotum was noted in 4/5 children

Anti-Müllerian Hormone (AMH); Congenital hypogonadotropic hypogonadism (CHH); Follicle-stimulating hormone (FSH); Gonadotropin-releasing hormone (GnRH); Human chorionic gonadotropin (HCG); Kallmann Syndrome (KS); Luteinizing hormone (LH); Multiple pituitary hormone deficiency (MPHD); Partial androgen insensitivity syndrome (PAIS); Septo-optic dysplasia (SOD); Subcutaneous (SC); Stretched Penile Length (SPL); Testicular Volume (TV).

## Data Availability

No new data were created or analyzed in this study.
